# Protein phosphatase 2A (PP2A): a key phosphatase in the progression of chronic obstructive pulmonary disease (COPD) to lung cancer

**DOI:** 10.1186/s12931-019-1192-x

**Published:** 2019-10-17

**Authors:** Cassandra P. Nader, Aylin Cidem, Nicole M. Verrills, Alaina J. Ammit

**Affiliations:** 1 Woolcock Emphysema Centre, Woolcock Institute of Medical Research, University of Sydney, Sydney, NSW Australia; 2 School of Biomedical Sciences and Pharmacy, Faculty of Health and Medicine, University of Newcastle, Callaghan, NSW 2308 Australia; 3 Priority Research Centre for Cancer Research, Innovation & Translation, Faculty of Health & Medicine, Hunter Medical Research Institute, New Lambton Heights, NSW 2305 Australia; 4 School of Life Sciences, Faculty of Science, University of Technology Sydney, Sydney, NSW Australia

**Keywords:** CIP2A, COPD, Inflammation, Lung Cancer, PP2A, SET

## Abstract

Lung cancer (LC) has the highest relative risk of development as a comorbidity of chronic obstructive pulmonary disease (COPD). The molecular mechanisms that mediate chronic inflammation and lung function impairment in COPD have been identified in LC. This suggests the two diseases are more linked than once thought. Emerging data in relation to a key phosphatase, protein phosphatase 2A (PP2A), and its regulatory role in inflammatory and tumour suppression in both disease settings suggests that it may be critical in the progression of COPD to LC. In this review, we uncover the importance of the functional and active PP2A holoenzyme in the context of both diseases. We describe PP2A inactivation via direct and indirect means and explore the actions of two key PP2A endogenous inhibitors, cancerous inhibitor of PP2A (CIP2A) and inhibitor 2 of PP2A (SET), and the role they play in COPD and LC. We explain how dysregulation of PP2A in COPD creates a favourable inflammatory micro-environment and promotes the initiation and progression of tumour pathogenesis. Finally, we highlight PP2A as a druggable target in the treatment of COPD and LC and demonstrate the potential of PP2A re-activation as a strategy to halt COPD disease progression to LC. Although further studies are required to elucidate if PP2A activity in COPD is a causal link for LC progression, studies focused on the potential of PP2A reactivating agents to reduce the risk of LC formation in COPD patients will be pivotal in improving clinical outcomes for both COPD and LC patients in the future.

## Introduction

Chronic obstructive pulmonary disease (COPD) is the third leading cause of death worldwide resulting in 3.5 million deaths annually, and globally affecting more than 200 million people [1]. The disease is characterised by two main respiratory conditions; chronic bronchitis and emphysema, and is characterised by severe inflammation in the lower airways. Although conventional treatment of COPD provide symptomatic relief of the disease, current medicines are unable to significantly halt disease progression. There is an increasing recognition of the overlapping prevalence of COPD with lung cancer (LC). Late stage cases of COPD have been identified to have a higher rate of disease progression to LC [2–4]. LC is one of the leading causes of mortality worldwide, accounting for more than 1.3 million deaths annually [5]. LC usually originates from the basal epithelial cells of the lung and is classified into two categories, non-small cell lung cancer (NSCLC) and small cell lung cancer (SCLC). NSCLC being the most prominent form of LC, accounts for approximately 85% of reported LC cases [6]. The current average 5-year survival rate of LC patients is 18.6% and is significantly lower than other leading forms of cancer [7]. The prevalence of LC is much greater in COPD patients compared to non-COPD individuals [2–4]. Accordingly to ARCTIC, a retrospective cohort study including over 100,000 participants, LC has the highest relative risk of development as a comorbidity of COPD; COPD patients are almost six times more likely to be diagnosed with LC than non-COPD patients [4]. An early study following COPD patients for a period of 10 years showed approximately 1% of patients with COPD develop LC per year [8]. More recently, a higher incidence of LC was reported with approximately 8.5% of COPD patients developing LC over a median follow up period of 5 years [2]. Moreover, prior to the development of any cancerous tumour, 40-70% of LC patients are diagnosed with COPD [2].

The high prevalence of COPD in people with LC suggests that there are common underlying molecular mechanisms between the two diseases. The most evident link between these two conditions is the commonality of smoking as the major etiological risk factor [9]. However, smoking is not the only causal link between the two conditions, as COPD is an independent risk factor for LC, irrespective of the smoking status of the individual [10, 11]. Common COPD related measures, including visual emphysema, airflow obstruction and respiratory exacerbations, are all independently indicative of LC [12]. Emphysematous COPD is of particular concern, as the prognosis of the cancer is worse when compared to non-emphysematous COPD [9–11]. Regardless of the staging of the cancer, tumour growth is most evident in areas of visual emphysema [13].

Given this clear clinical link, a great deal of research has focused on uncovering the molecular mechanisms that lead to LC initiation. Cancer development and progression in any cell type is often due to a combination of intrinsic factors; such as genetic alteration, and extrinsic factors; such as environmental exposure [14]. COPD and LC are heterogeneous by nature, thus it is likely that intrinsic and extrinsic factors do not act as separate entities to cause disease, but rather play co-dependent causative roles [15]. Similarly, it is likely that multiple molecular interactions contribute to this disease progression. A number of putative molecular insults, including excessive inflammation and reactive oxygen species, as well as perturbed cellular senescence, have been proposed as the underlying molecular links between COPD and LC (reviewed in [3, 9, 15–21]). To date however, no review has explored the potential role of protein phosphatase 2A (PP2A) in the progression of COPD to LC. In this review therefore, we explore how: the surrounding molecular interactions in the disease setting induce PP2A inactivation and may result in a predisposition of COPD patients to the development of LC; and highlight how targeting PP2A activation therapeutically could prove beneficial in not only controlling COPD but also to limit the progression to LC.

## Structure and biochemistry of PP2A

PP2A is involved in many regular cell functions, making up 1% of total cellular protein and accounting for the majority of serine threonine phosphatase activity in mammalian cells [22, 23]. It is responsible for approximately 30-50% of all serine threonine phosphatase activity in many cell types [24]. PP2A regulates a range of cell processes including metabolism, growth, apoptosis and cell cycle progression [25, 26]. PP2A exists as a holoenzyme composed of three primary subunits – A, B and C [27]. The scaffolding (A) and catalytic (C) subunits are ubiquitously expressed and form a catalytically active heterodimer. The scaffolding A subunit consists of two main isoforms; Aα and Aβ [26]. There are four families of the regulatory (B) subunit, each with multiple isoforms, and these display cell and tissue-specific expression [23]. The catalytic C subunit also exits in two isoforms; Cα and Cβ [26]. The addition of the regulatory B subunit to the core A-C dimer provides sub-cellular targeting and substrate specificity [27].

## Mechanisms responsible for PP2A inactivation

PP2A is a ubiquitous phosphatase responsible for maintaining cellular function. Thus, it is not surprising that genomic or non-genomic disease mechanisms that impact on PP2A functionality result in unwanted consequences. Recent studies have demonstrated negative disease implications resulting from PP2A inhibition in COPD [28–32]. PP2A inactivation is also known to play an important role in LC, understandable given that PP2A is a well-established tumour suppressor [25, 33–36]. Thus, it is plausible that PP2A could be a key molecular link in progression of COPD to LC and a greater understanding of the molecular mechanisms responsible for PP2A inactivation in these disease settings is vital. These can be direct or indirect and will discussed below (and tabulated in Table 1).

**Table 1:** Summary of PP2A related signalling pathways in lung disease

	PP2A Interaction	Upstream	Downstream	Physiological effect	Disease	Ref.
Inhibitory						
CIP2A	Directly inhibits PP2A	**↑**EGFR	**↓**ERK **↑**MMP1 and MMP9	ECM degradation	COPD, Lung Cancer	[28, 82]
			**↓**ELK-1 **↑**AKT	Cell survival	Lung Cancer	[94, 95, 98, 104, 106]
			**↑**c-MYC	Proliferation, self-renewal and oncogenic transformation	Lung Cancer	[70, 95, 99, 103]
			**↑**MKK4, JNK, ATF2, c-jun	Proliferation	Lung Cancer	[36]
SET	Directly inhibits PP2A		**↑**AKT, cyclin D1, MMP9 **↓**P27	Proliferation, ECM degradation and invasion	Lung Cancer	[34, 35]
		**↑**PCDH7	**↑**ERK, cyclin D1, MMP9 **↓**P27	Proliferation, ECM degradation and invasion	Lung Cancer	[34, 35, 83]
			**↑**c-MYC **↓**NRDG1, RIPK1	Invasion, oncogenic transformation and metastasis	Lung Cancer	[34, 113]
PME-1	Stablises inactive PP2A		**↑**ERK, AKT	Proliferation	Lung Cancer	[81]
MID1	Degrades PP2A-C	**↑**TRAIL		Fibrosis, collagen deposition, cell survival and proliferation	Asthma, Pulmonary fibrosis, Lung cancer	[116, 117]
Beneficial						
Calpains	PP2A inactivates calpains		**↓**μ - and m-calpains	Suppression of invasion and migration, and wound healing	Lung Cancer	[123, 124]
Rho B	Rho B binds to PP2A-B55 to increase active PP2A	↑RASSF1A	**↓**AKT-1 **↑**GEF-H1	suppression of invasion and migration	Lung Cancer	[125, 126] [128]
TTP	PP2A activates TTP	**↓**P38- MAPK, MK2	**↓**IL-6, IL-8 **↓**LATS2, cyclin B1	Suppression of inflammation and proliferation.	COPD, Asthma, Lung cancer	[29–31]

### Mechanisms that directly inactivate PP2A: genetic factors

#### COPD

A key genetic risk factor in COPD is alpha-1-antitrypsin (A1AT), a serine protease inhibitor involved in protecting the lung from destructive protease actions, particularly that of neutrophil elastase enzymes [37]. A1AT mutations can result in the inactivation of PP2A and result in subsequent respiratory inflammation. A1AT is encoded in humans by the SERPINA1 gene, with severe A1AT deficiency most commonly caused due to homozygosity of the Z allele [37, 38]. A1AT deficiency is a hereditary disorder associated with severe pulmonary emphysema and thus a high risk of COPD development [39–41]. PP2A and A1AT regulate similar biological processes that play a key role in COPD. Studies have shown the multifunctional role of A1AT in anti-inflammatory and immuno-regulatory functions in cellular and mouse models of airway respiratory disease [42–44]. Exogenous human A1AT therapy was shown to suppress tumour necrosis factor (TNF) related smoke-induced inflammation and ameliorate matrix breakdown of the lung epithelium, providing partial protection against emphysema in mice [43]. A study from a large population-based database has shown the prevalence of COPD and emphysema in A1AT deficiency was 32 and 21%, respectively [45]. A1AT deficiency has been linked with increased severity of symptoms of COPD [45]. Patients with A1AT deficiency have more frequent medical consultations and longer hospitalisations when compared with non-A1AT deficient COPD subjects [45]. The development of COPD in A1AT deficient patients has been clinically linked with alveoli and small airway structural breakdown caused by protease expression, apoptosis and heightened inflammatory response [42, 43, 46].

Importantly, A1AT deficiency is linked with the inhibition of PP2A in COPD. A1AT deficient COPD patients have been found to have significantly lower levels of active PP2A in their neutrophils in comparison to COPD patients without the deficiency [47]. Notably, administration of high dose (120 mg/kg) functional A1AT protein augmentation therapy to A1AT deficient individuals increases active PP2A levels in lung epithelial cells and alveolar macrophages [47]. The increased level of PP2A activity was further associated with the downstream repression of pro-inflammatory cytokine levels and protease activity within the lung epithelium of extracted subject cell samples [47]. The molecular mechanisms have been delineated; functional A1AT is regulated by protein tyrosine phosphatase 1B (PTP1B) [47, 48]. A1AT increases PP2A activity in the lung by triggering PTP1B to dephosphorylate PP2A-C subunit at tyrosine site 307 [47]. Previous studies have demonstrated the anti-inflammatory role of PP2A in inhibiting the activation of the TNF pathway and A1AT therapy was shown to inhibit the pro-inflammatory TNF signalling in A1AT deficient neutrophils [44]. These studies show that functionally active A1AT protein is an important factor for PP2A activation in COPD patients. Targeting PP2A reactivation may be an effective therapeutic strategy in A1AT-deficient COPD patients.

#### Lung Cancer

Many studies have established a role for PP2A as a tumour suppressor, and hence disruption of the PP2A subunit/s and/or the function of the PP2A trimer, is established as a contributing factor in the development of many cancers [25, 33–36]. Inactivation of PP2A may be due to somatic mutations, loss of heterozygosity and/or altered expression of individual subunits, as well as increased expression of PP2A inhibitory proteins (see below). These associations result in the disruption of the PP2A enzyme complex and functionality through preventing the core PP2A-A subunit from binding to the B and/or C subunits [49, 50].

Genetic mutations have been detected in all subunits of PP2A in cancer [51–53]. Cancer associated mutations of the A subunits are defective in binding the B and/or C subunits of PP2A [51–53]. Analysis of The Cancer Genome Atlas (TCGA) data indicates that mutations to PP2A subunit genes are relatively rare in lung adenocarcinoma. Notably, the most recurrent LC related PP2A mutations are found in the *PPP2R1A* gene, encoding for the PP2A-Aα subunit, and occurring in 1.3% of tumours [50, 54]. These mutations are functionally important as knock-in mice with a LC associated mutation in PP2A-Aα (E64D) resulted in the suppression of PP2A function, and increased incidence of benzoprene-induced LC by 50 to 60% [50]. PP2A-Aα-E64D mutation further enhanced tumour formation in K-Ras-G12D driven mouse LC [54]. These findings further suggest that the functional PP2A holoenzyme is important in tumour suppression and PP2A-Aα subunit mutations may play an essential role in tumourigenesis of the lung [50, 54].

In vitro studies have also demonstrated cancer-associated PP2A-Aβ mutations induce impaired binding to the B and C subunits [49, 55]. PP2A-Aβ mutations alter the associations with other PP2A subunits, much like PP2A-Aα mutations [49, 55]. Mutation of the PP2A-Aβ subunit results in the dysfunction of GTPase RalA, a protein involved in transcription, migration, transport, apoptosis, and cell proliferation [51]. Normal function of PP2A-Aβ binds and regulates the activity of GTPase RalA protein via phosphorylation [51]. Dephosphorylation of RalA leads to the subsequent inactivation of RalA, and of PP2A-Aβ losing its regulatory tumour suppressive capabilities, resulting in failure to reverse tumourigenic phenotypes and cellular dysfunction [51, 56].

It is also noteworthy to mention inactivation of PP2A caused by possible germline mutations. Mutations in the gene encoding the PP2A-Aβ subunit (*PPP2R1A*) have been identified in lung tumours and LC-derived cell lines [51]. In one LC cell line, H2009, the mutation was also found in a matched lymphoblastoid cell line, suggesting it was a germline mutation. The resulting amino acid substitution, G90D, was subsequently found to inhibit binding to the tumour suppressive PP2A-56γ subunit [57]. Germline mutations and/or SNPs in PP2A subunit genes have more recently been identified in association with mental retardation [58, 59], however PP2A inactivation arising from germline mutations are yet to be comprehensively explored in the context of lung disease.

Mutations of genes encoding the regulatory subunits of PP2A have also been identified in lung adenocarcinomas [60]. The PP2A-B subunit is important in its regulation of functional cell proliferation and migration [61–63]. Functional PP2A-B56γ isoform dephosphorylates ERK, leading to inactivation of ERK and the inhibition of cell proliferation and migration [62, 63]. A later study suggested the inhibition of PP2A-B56γ is involved in sustained activation of ERK and subsequent lung adenocarcinoma cell proliferation [60]. Overexpression of PP2A-B56γ3 isoform in human LC cell lines partially reverses the tumourgenicity of these cells via the cell proliferation suppression and reduction of colony numbers [61].

### Mechanisms indirectly inactivating PP2A: endogenous inhibitors (CIP2A and SET)

In cancerous diseases, non-genomic activity regulates PP2A to a much greater extent than direct genetic mutations [33]. PP2A activity is modulated by a number of endogenous proteins including acidic nuclear phosphoprotein 32 family members A [64], C [65] and E [66] (ANP32A, ANP32C and ANP32E), inhibitor 1 of PP2A (A1PP2A), inhibitor 2 of PP2A [67] (I2PP2A or SET), endosulfine alpha (ENSA) [68], protein phosphatase methylesterase-1 (PME-1) [69] and cancerous inhibitor of PP2A (CIP2A) [70].

ANP32A, also referred to as I1PP2A, acts as a tumour suppressor and ANP32C is oncogenic, with both aberrantly expressed in human malignancy [64, 65]. Their involvement has been reported in colorectal cancer [71], oral cancers [72], leukaemia [73], and prostate cancer [74], but neither have been reported to be pivotal in lung carcinogenesis.

ENSA is a phosphorylation-dependent inhibitor of PP2A, responsible for the regulation of mitosis, specifically inhibiting PP2A-B55 activity during the M phase [68]. Studies have shown the downregulation of the PP2A-B55 regulatory subunit in non-small cell lung carcinomas [75, 76], however, the underlying mechanism of ENSA and its role in PP2A modulation in lung disease remains poorly explored.

PME-1, a PP2A modulator, has been identified to have a role in the biogenesis of PP2A holoenzyme stability and functionality [77]. The upregulation of PME-1 has been shown to stabilise inactive PP2A through binding and demethylation of the PP2A-C subunit [69, 78]. The overexpression of PME-1 has been extensively studied in advanced stages of glioblastoma [79] and has been demonstrated to serve as a progression marker in late stage endometrial cancer [80]. Li et al., identified amplification of PME-1 gene (PPME1) expression in a small subset LC patient cohort (3.1%) [81]. The study showed PME-1 knockdown resulted in decreased PP2A-C demethylation and subsequent AKT and ERK phosphorylation in PPME1 amplified LC cells [81]. PME-1 was further found to be overexpressed in various KRAS-mutant LC cell lines when compared to human bronchial epithelial (HBE) cells [82]. PME-1 has also been identified to drive kinase inhibitor resistance in glioma cells. More recently, Kauko et al., have demonstrated the role of PME-1 in resistance to MEK inhibition in KRAS-mutant LC cells [82]. These studies highlight the importance of PME-1 and its role on PP2A activity modulation, however, further studies are required to better establish the significance of PME-1 in lung disease.

In contrast, SET and CIP2A have been extensively demonstrated as endogenous inhibitors of PP2A in the context of lung disease, therefore are discussed comprehensively below [26]. SET has been reported to interact with two subunits of PP2A, the PP2A-A subunit [83] and the PP2A-C subunit [84]. The interaction of CIP2A is a little more enigmatic; however, it appears to exert its effect by interacting with PP2A-B [85].

#### CIP2A in COPD

Recently, Nath et al. identified that both the mRNA and protein expression of CIP2A is increased in HBE cells of COPD patients compared to cells from non-smokers [28]. Further studies showed emphysematous changes in mice exposed to chronic cigarette smoke correlated with increased CIP2A expression and reduced PP2A activity [28]. Importantly, CIP2A was the only PP2A inhibitor induced by chronic cigarette smoke exposure [28]. Silencing CIP2A via siRNA resulted in increased PP2A activity in HBE cells from COPD patients, highlighting the regulatory control of PP2A activity by CIP2A in the context of COPD [28]. Interestingly, a prior study reported increases in PP2A activity in samples from human subjects with emphysema, although no increase in the total PP2A-C protein was observed compared to age matched healthy controls [86].

CIP2A dysregulation is also observed in smokers without COPD, but notably the increase in CIP2A expression in COPD patients is greater than that in smokers without COPD [28]. This suggests that CIP2A overexpression and its downstream regulation of PP2A are key factors in the development of COPD rather than smoking alone.

In addition, epidermal growth factor receptor (EGFR) response and phosphorylation is increased in COPD, resulting in the elevation of CIP2A expression [28]. Treatment of HBE cells from COPD patients with Erlotinib, a known EGFR inhibitor, increased PP2A activity, downregulating CIP2A mRNA and protein, resulting in decreased ERK phosphorylation and expression of matrix metalloproteinase 1 (MMP1) and MMP9 compared to non-COPD subjects [28]. MMPs degrade the extracellular matrix (ECM) and promote tissue damage by activating cytokines such as TNF [87]. Collectively this worsens the state of inflammation and cellular destruction characteristic of COPD pathogenesis. Importantly, MMPs are also biologically relevant to cancer progression, as they are involved in the proteolytic degradation of ECM and cell-ECM interactions which allow invasion and metastasis [88]. These similarities highlight how PP2A and its regulation by CIP2A may in fact be a mechanistic link in progressing COPD towards a more tumourigenic phenotype. Although, further studies are required to explore CIP2A activity in COPD patients to validate findings by Nath et al. and extrapolate the potential of CIP2A-PP2A activity in the progression to LC, possibly through clinical longitudinal studies.

#### CIP2A in Lung Cancer

CIP2A is one of the most common oncoproteins in human malignancy, with approximately 70% of tumours reported to overexpress CIP2A [70, 89, 90]. These patterns are no different in LC [36, 91], with one study finding that 63% of LC tumours express elevated CIP2A protein compared to para-tumour normal tissue [92]. Increased CIP2A expression also predicts poorer clinical outcomes in NSCLC patients [93–95]. CIP2A mediated inhibition of PP2A impairs its crucial role in tumour suppression, as it can no longer inactivate numerous kinase-driven intracellular signalling pathways that allow cancer progression [89].

The AKT pathway is activated in many LCs to promote cell survival [92, 96, 97]. Studies in LC cell lines have shown that inhibition or degradation of CIP2A remarkably reduces phosphorylated, and hence active, AKT (p-AKT) [95, 98]. The decrease in p-AKT induces inhibition of cell proliferation and increases apoptotic cell death [98]. Similarly, the oncogenic transcription factor, c-MYC signalling plays a major role in tumourigenesis, and is well described to be regulated by PP2A, specifically being de-phosphorylated at Serine 62 [99]. Work by Arnold and Sears identified that when PP2A is bound to the B56α residue specifically, it directly dephosphorylates c-MYC at serine 62 [100]. In later studies the PP2A-B56α complex was shown to be active in c-MYC regulation in malignancies [101, 102]. Increased CIP2A levels in human malignancy prevent c-MYC dephosphorylation by PP2A, and thus stabilises c-MYC to promote proliferation, self-renewal and oncogenic transformation [70, 99, 103]. This has been demonstrated in LC cells where CIP2A inhibition results in decreased c-MYC protein levels, reduced proliferative capacity and increased apoptosis [95]. Additionally, CIP2A has been demonstrated to regulate the MKK4/JNK signalling pathway in LC cells to promote cell proliferation [36]. CIP2A knockdown decreases JNK phosphorylation, and downstream transcription factors such as ATF2 and c-jun, while CIP2A overexpression increases MKK4, the upstream kinase of JNK [36]. However, inhibiting JNK alone does not account for the entirety of CIP2A induced cell proliferation in LC cells [36], suggesting that a combination of JNK, c-MYC, AKT and possibly other pathways are important in the oncogenic effects of CIP2A in LC.

Collectively, the sustained CIP2A-mediated inhibition of PP2A promotes a number of signalling pathways that progress the pathogenesis of LC (as described above), and suggests therapeutically targeting CIP2A/PP2A in LC will have merit. This argument is supported by the discovery that a number of established anticancer drugs appear to act in a CIP2A/PP2A dependant manner [104]. As seen in COPD, EGFR appears to regulate CIP2A expression. Afatanib and Erlotinib are EGFR tyrosine kinase inhibitors (TKIs), commonly used to treat NSCLC patients with EGFR mutations [105]. While specifically developed for mutant EGFR, both also show efficacy in some patients with non-EGFR mutations. This may be via the suppression of CIP2A, as treating NSCLC cell lines without EGFR mutations with these TKIs and derivatives like TD-19 was associated with the down regulation of CIP2A [94, 104, 106]. This subsequently increased PP2A activity, decreased p-AKT and, increased apoptosis of cancerous cells [94, 104, 106]. One particular study demonstrated that Afatanib decreases the transcription of CIP2A through a reduction in the Elk-1 transcription factor binding to the promoter region of CIP2A [94]. This study also demonstrated the same CIP2A-PP2A-AKT cascade in NSCLC xenografts treated with Afatanib and showed that in clinical samples from NSCLC resections CIP2A expression predicted poor prognosis and was correlated with Elk-1 expression [94]. Consistent with these findings, Kauko et al. found that PP2A inhibition induces TKI resistance in KRAS mutant lung cancer [82]. CIP2A depletion was able to sensitise the cells to TKIs targeted at MEK and EGFR pathways [82]. Anti-cancer treatment through the CIP2A-PP2A-AKT cascade has more recently been demonstrated through the treatment of TKI resistant NSCLC with Cucurbitacin B, a plant based therapeutic [107]. Treatment with Cucurbitacin B results in downregulation of the CIP2A expression increases in PP2A activity and decreased phosphorylated AKT, in combination with the degradation of EGFR, collectively increasing the apoptosis of the cancerous cells [107]. Whilst these studies begin to elucidate how CIP2A expression is regulated by EGFR inhibitors and similar therapeutics, further studies are required, and more importantly further studies are necessary to determine whether a reduction in CIP2A expression correlates with the beneficial response of these treatments in LC patients.

#### SET in COPD

Research has yet to be conducted on the PP2A inhibiting role of SET in COPD. Given PP2A activity is inhibited in COPD it would be of interest to investigate if this is mediated, at least in part, by increased SET expression, and thus may provide a further understanding of the mechanistic link between COPD and LC progression.

#### SET in Lung Cancer

Whilst insufficient research addresses the role of SET in COPD, SET has been explored extensively in cancerous tissue. SET is a well-established oncogene in a number of different human malignancies [73, 108–111]. LC is no exception, with SET identified to be overexpressed in NSCLC, adenocarcinoma and LC cell lines [34, 35, 112]. SET expression has also been shown to positively correlate with higher clinical stage at diagnosis, lymph node metastasis, and poorer prognosis [35].

SET plays an important role in altering PP2A regulated oncogenic signalling pathways, promoting the growth and progression of cancer cells [67]. Like CIP2A, SET regulates AKT and ERK signalling. Knock-down or antagonism of SET in LC cell lines directly increases PP2A activity, and results in decreased phosphorylated AKT and ERK [34]. This decreases proliferation tumour sphere formation, and invasion of A549 LC cells [34, 35]. When SET is active, AKT and ERK signalling are stimulated, increasing cyclin D1 and decreasing p27, key regulators of the cell cycle. SET expression also increased secretion of MMP9 to promote invasiveness [35]. Upstream of these pathways, SET-PP2A complex formation is increased by the cell surface receptor protocadherin 7 (PCDH7) which is upregulated in NSCLC [83].

In addition, SET plays a role in the epithelial to mesenchymal transition (EMT), a key hallmark of cancer invasion and metastasis [34, 113]. The role of SET in this context is largely through interaction with c-MYC [113]. As mentioned in detail above in the CIP2A in LC section, PP2A dephosphorylates c-MYC at Ser 62, therefore allowing it to be targeted for degradation, reducing oncogenic signalling [24, 99, 100]. In Cisplatin-sensitive and Cisplatin-resistant A549 LC cells, SET inhibits PP2A activity therefore stabilising c-MYC which acts to downregulate NRDG1, a metastasis suppressive protein [113]. When SET is inhibited in these cells, PP2A is activated, EMT is reversed, chemo-sensitivity to cisplatin in restored, and in vivo tumour growth and metastasis is reduced [34, 113]. The restoration of chemo sensitivity to Cisplatin is likely a consequence of reduced EMT as the acquisition of a mesenchymal phenotype contributes to chemotherapy resistance [114]. The importance of SET in lung tumorigenesis is further highlighted with the use of FTY720 (Fingolimod), a known inhibitor of SET [112]. FTY720 treatment is able to increase cell death through RIPK1 dependent necroptosis, and suppress the growth of A549 xenografts in mice [112]. In further experimentation it will be important to establish the relationship of SET- PP2A activity in samples from LC patients rather than solely cell lines and xenograft models. Collectively, the prominent involvement of both endogenous inhibitors, SET and CIP2A in LC pathophysiology emphasises the importance of PP2A as a key regulator in this disease state.

## Notable PP2A-mediated pathways common to both COPD and lung cancer

Other molecular interactions involving PP2A have been demonstrated to be common between chronic inflammatory respiratory disease and LC. Whether the molecular pathways result in direct or indirect inactivation of PP2A is unknown at present and warrants further investigation. However, in some studies, activation/re-activation of PP2A by novel PP2A activators has resulted in beneficial effects.

### MID1

Midline 1 (MID1) is an E3 ubiquitin ligase that associates with microtubules, and blocks the accumulation of PP2A-C by targeting it for proteasomal degradation [115]. MID1 has been shown to mediate PP2A inhibition in asthma mice models [116], and more recently in pulmonary fibrosis, in mice models and biopsy tissue [117]. In these models, tumour necrosis factor-related apoptosis-inducing ligand (TRAIL) upregulates MID1 expression to negatively regulate PP2A activity and thereby the dephosphorylation of downstream pro-inflammatory molecules [117]. The activation of PP2A reversed the TRAIL/MID1/PP2A cascade and showed evident improvements in lung function and reduced fibrotic changes such as collagen deposition [117]. Likewise, the negative regulation of PP2A by MID1 has been displayed in LC [118]. MID1 is upregulated in LC cell lines, compared bronchial epithelial cells, and in lung adenocarcinoma tissue, compared to normal adjacent tissue [119]. PP2A is inversely related to MID1, being downregulated in the respective LC cells and tissue [119]. The transfection of lung adenocarcinoma cell lines with MID1 siRNA, upregulated PP2A expression and induced apoptosis and cell cycle arrest [119]. Activating PP2A via FTY720 also prompted apoptosis and cell cycle arrest [119]. Possibly related to this activity of MID1 in lung cancer is that of α4, a regulatory protein of PP2Ac. α4 has been reported to be highly expressed in 84% of primary lung cancers [120], and overexpressed in lung adenocarcinoma [121]. During PP2A biogenesis α4 binds and thus sequesters PP2Ac, and inhibits proteasomal degradation by MID1 prior to the formation of the PP2A complex [122]. Whilst α4 inhibits MID1, it also possesses an unstructured and flexible C terminal domain with the ability to bind MID1. In cancerous phenotypes it is plausible that the regulatory role of α4 alters and in fact binding to PP2Ac displaces it from A and B subunits, and in doing so brings PP2Ac into closer proximity with MID1 promoting proteasomal degradation [77, 120]. Collectively, these studies highlight the importance of MID1/PP2A for the control of both inflammatory related pathways and cell cycle control in both COPD and LC.

### Calpains

Calpains are important mediators of metastasis in LC [123]. PP2A inhibition, either via the SV40 small T or RNAi directed to the PP2A-C subunit, has been shown to promote calpain phosphorylation, increasing calpain activity and associated cell migration and invasion [123, 124]. PP2A directly dephosphorylates μ - and m-calpains in vitro, and overexpression of PP2A-C suppressed nicotine-stimulated phosphorylation of μ - and m –calpains, inhibiting calpain activity, wound healing and cell migration [124]. These findings suggest the function of PP2A as a calpain phosphatase, inactivating μ- and m-calpains via dephosphorylation and subsequent suppression of invasion and migration of human LC cells [124].

### RhoB

The expression of RAS homolog gene family, member B (RhoB), is also linked to the suppression of migration and invasion of LC cells [125, 126]. The regulation of migratory and invasive properties of LC cells via RhoB has been shown to be regulated by its interaction with PP2A [127]. RhoB regulates PP2A activity via binding to the PP2A-B55 regulatory subunit, suggesting the inhibition of the AKT1 pathway [127]. Inhibition of RhoB in bronchial cells using siRNA significantly reduced PP2A activity and in contrast, the overexpression of RhoB induced a significant increase in activated PP2A [127]. Similarly, the mechanism of a common tumour suppressor gene, RASSF1A, suppresses invasion and metastasis in NSCLC cell lines through PP2A/RhoB and guanine nucleotide exchange factor H1 (GEF-H1) activity [128]. The depletion of RASSF1A by knockdown studies resulted in decreases in PP2A activity and decreased the activation of GEF-H1 [128]. Subsequent knockdown and immunoprecipitation studies revealed that RASSF1A likely exerts its tumour suppressor effect by PP2A mediated dephosphorylation of GEF-H1 allowing subsequent stimulation of RhoB [128]. Collectively, this suggests the importance of reduced RhoB expression in LC in understanding mechanisms that impair PP2A activity, allowing for oncogene-transforming properties [127].

### TTP

**Fig. 1:**
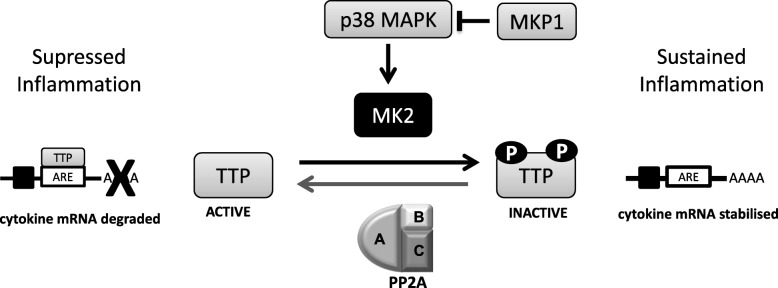
p38 MAPK inflammatory signalling is regulated by PP2A. p38 MAPK activates MK2, subsequently mediating the phosphorylation and thereby the inactivation of TTP. Inactive TTP allows for the stabilisation of pro-inflammatory cytokine mRNA and thus sustained inflammation. In response to sustained inflammation, MKP-1 acts as a negative feedback effector, dephosphorylating and thereby inactivating p38 MAPK, and inhibiting downstream pro-inflammatory signalling. PP2A dephosphorylates and activates TTP. Active TTP degrades pro-inflammatory cytokine mRNA by binding to the ARE 3′ untranslated region, resulting in the suppression of inflammation

PP2A plays a major role in the dynamic control of p38 mitogen activated protein kinase (MAPK) mediated inflammation during severe asthma and COPD [29, 129]. In COPD, the abundance of phosphorylated p38 MAPK positive cells increases in the alveolar region, subsequently promoting the p38 MAPK inflammatory pathway [130]. p38 MAPK acts through MAPK kinase 2 (MK2) to phosphorylate and thus inactivate tristetraprolin (TTP) [131, 132]. TTP is an early response gene that binds to adenosine/uridine rich elements (ARE) at the 3′ untranslated region of genes involved in the pro-inflammatory response, deadenylating and degrading the mRNA [133]. When TTP is phosphorylated by MK2, it exists in an inactive state, therefore pro-inflammatory mRNA is stabilised and inflammation is sustained [134]. The dephosphorylation of TTP by PP2A activates TTP, repressing inflammatory cytokine secretion (Fig. 1). The temporal regulation of this cascade (and dynamic control in concert with MKP-1) has been demonstrated in a number of respiratory cell lines via the use of TNF induced inflammation to mimic the setting of COPD [29–31]. In airway smooth muscle cells, TNF induces TTP expression in a p38 MAPK dependent manner, as early as 30 min after stimulation, directly increasing the secretion of IL-6 [29]. In broncho-epithelial and NSCLC cells lines, TNF induces both IL-6 and IL-8 secretion, and inhibition of PP2A additively enhances the secretion of these cytokines, which collaboratively promote an inflammatory environment [30, 31]. The opposite approach has also been studied, in which well-defined PP2A activators were shown to result in the dephosphorylation of TTP and decreased pro-inflammatory cytokine secretion [30–32]. The regulation of this pathway is likely shifted to favour the phosphorylation and thus inactive form of TTP in the context of COPD and severe asthma [30]. Therefore, targeting the activation of PP2A to compensate for this equilibrium shift could enhance TTP activation and counteract chronic inflammatory mediated disease progression.

Notably, TTP also plays a role in lung cancer. TTP functions as a tumour suppressor gene via mRNA degradation and thus down regulation of mass critical cancer genes [135]. TTP expression has been shown to be decreased in multiple human malignancies [136–138]. Fallahi et al. determined a ‘low-TTP’ gene signature of 50 genes from the analysis of a number of cancer types form TCGA [139]. According to Ingenuity Pathway Analysis (IPA) software, 80% of pathways altered by TTP in this gene signature are inflammatory pathways, and more specifically associated with innate immunity [139]. This is consistent with the role of TTP in COPD and other inflammatory disease states [135, 136]. In LC cell lines, levels of TTP transcripts are comparable to normal lung tissue, excluding that of A549 NSCLC cells in which TTP mRNA is barely detectable [137]. This low expression in A549 cells was confirmed in a subsequent study, in addition to highlighting significantly lower TTP mRNA expression in patient derived lung tumour tissue compared to patient matched non transformed tissue [138]. From the analysis of TCGA, patients with low TTP expressing lung adenocarcinomas have more aggressive tumours with increased necrosis and decreased survival rates compared to patients with high TTP expressing tumours [139]. Thus far, two specific mRNA targets of TTP have been identified in A549 NSCLC cells, large tumour suppressor kinase 2 (LATS2) [140], and cyclin B1 [141]. LATS2 and Cyclin B1 are important for cell cycle progression, survival and proliferation [140, 141]. Currently, studies on the role of TTP in LC are very limited. Given the prominent role of TTP in COPD, it would be important to determine if TTP activity in the context of LC is similar to that of COPD, and thus determine if TTP activity downstream of PP2A contributes to the predisposition of COPD patients to LC.

## PP2A inactivation creates a favourable microenvironment for lung cancer progression

The most reported biological hallmark of COPD that predisposes the development of LC is chronic inflammation and associated proteolytic activity [15, 17, 21]. The combination of persistent pro-inflammatory cytokine release and, MMP and cathepsin (CTS)-mediated degradation of the ECM, creates a tissue niche that promotes key hallmarks of cancer such as ECM remodelling, EMT and angiogenesis [15].

As outlined above, p38 MAPK, through its inactivation of TTP, is the most effective MAPK for stabilising the mRNA of pro-inflammatory cytokines and chemokines relevant to COPD inflammation. TTP regulates a number of cytokines that promote inflammatory processes, including that of TNF, IL-6, IL-8, IL-17 and IL-33 [142–146]. Each of these cytokines play slightly different roles, but collectively promote an inflammatory milieu [142–146]. The cytokine secretion results in the recruitment of neutrophils and inflammatory monocytes, which infiltrate the lower airways of the lung leading to respiratory exacerbations and sustained inflammation [147]. Chronic inflammation promotes cell turnover, increasing the susceptibility to genetic error, in addition to promoting EMT, collectively stimulating LC initiation [21].

Chronic inflammation also alters the micro-environment and encourages LC formation through promoting the activation of MMPs and CTSs to promote a cascade of proteolytic degradation [148, 149]. Elastin and collagen are both degraded, but typically it is the loss of elastic fibres in the parenchyma of the lung that leads to the enlargement of peripheral airspaces in the distal and terminal bronchioles [150, 151]. MMPs are one of the greatest contributors to ECM degradation in COPD, in particular in emphysematous COPD [15]. MMP9 and MMP12 are highly involved in elastolysis, thus they are reported as two of the key MMPs active in COPD [87]. In fact, it is MMP9 that accounts for the majority of elastolytic activity from alveolar macrophages in patients with COPD [152]. These two MMPs and others have been extensively reviewed in the general context of COPD by Navratilova and colleagues [148].

Importantly, MMP activation in COPD is regulated through p38 MAPK signalling and subsequent PP2A interactions. When purified PP2A is introduced intra-tracheally into the lungs of mice with smoke induced inflammation, PP2A activity is increased and MMP9 expression is decreased [86]. Even though this study does not specify the degradation of MMP9 mRNA by TTP, it is very plausible as MMP9 has been shown to be regulated by TTP in an direct manner in head and neck cancer [144]. MMP12 is likely regulated by p38 MAPK, as it is has been shown to be induced by cigarette smoke and subsequent TNF signalling contributing to the progression of emphysema [153].

Similar to MMPs, CTS activity acts in similar manner to degrade ECM proteins, and thus contribute to COPD pathogenesis [154]. CTSS [155] CTSG [156], and CTSE [157] levels are increased in the serum of COPD patients compared to healthy control patients. Both CTSE and CTSS are negatively correlated with the severity of airflow limitation [155, 157], and CTSS is also negatively related to the severity of emphysema [155]. The specific contribution of these different CTSs and others in COPD has extensively reviewed by Dey et al. [154].

Most relevant to this review, CTSS has very recently been linked to PP2A activity in mouse models of COPD and in human COPD tissue [158]. CTSS expression and activity is induced in wild type mice exposed to chronic cigarette smoke. CTSS knock out mice with identical exposure are resistant to inflammation, airway hyperresponsiveness, airspace enlargement and loss of lung function that is seen in the wild type condition [158]. Importantly, the expression and activity of CTSS is greater in isolated HBE cells of COPD patients compared to non-smokers and this activity is negatively regulated by activating PP2A [158]. Similarly, enhancing PP2A activity prevented chronic smoke-induced COPD like symptoms in wild type mice [158]. Doherty et al. also observed reduced ERK phosphorylation when PP2A was active, suggesting partial upstream regulation by ERK signalling pathways [158]. Whether or not PP2A activity is also regulated by p38 MAPK in this context is yet to be investigated, although very possible, as cathepsin activity has been shown to be regulated in part by p38 MAPK in chondrocytes [159]. Doherty’s study is the first to elucidate the link of cathepsin activity to PP2A in the context of COPD, although as highlighted by Janga & Hamm-Alvarez, this study highlights CTSS and PP2A regulation in smoking induced COPD, so further studies must be conducted in smoking independent COPD models to validate these results [160]. Further studies should also be conducted to determine if other CTS activity is regulated by PP2A in the context of COPD.

Collectively, the inflammatory and proteolytic environment promotes EMT. EMT is the process by which epithelial cells change in cell polarity, lose cell-cell interactions and gain a more invasive and migratory mesenchymal phenotype [114]. EMT undoubtedly contributes to tumour initiation and metastasis; however whether EMT is an essential process for cancer initiation and progression is a topic of much debate [161, 162]. The induction of airway inflammation by either repeated cigarette smoke exposure or TNF leads to the development of LC in mice [163, 164] and inflammation induced by cigarette smoke has been shown to induce EMT in pulmonary epithelial cells and the airways of mice [165]. Similarly EMT has been suggested to be active in COPD patients, both in the larger and smaller airways [114, 166]. Wang et al. demonstrated that in the small airway epithelium of COPD patients, E cadherin, a common epithelial cell marker, was decreased and vimentin, a marker of EMT, was significantly increased [166]. In a later study multiple markers of EMT including, S100A4, vimentin and MMP9, were all present in the bronchial epithelial layer and the reticular basement membrane (rbm) of airway biopsies from COPD patients [167]. In addition to these markers, rbm fragmentation was evident, suggesting the possibility of mesenchymal transformation and the migration of these cells through the rbm [114, 167]. In larger airways of COPD patients, rbm fragmentation is accompanied by increased VEGF secretion and hypervascularity, suggesting active angiogenesis [167, 168]. Importantly, when EMT is accompanied by increases in angiogenesis in the rbm and the epithelial cell layer a pro-cancer stroma is formed [169].

The activity of MMPs and CTSs, and thus alterations in the ECM during COPD greatly contributes to the development of LC, and in fact, both MMP and CTS activity is elevated in LC [170]. The ECM is a bioactive environment that has the potential to orchestrate and alter cellular responses [171]. During COPD, changes in the composition, rigidity and folding of ECM proteins in the lung amends cell interactions and signalling [171]. Therefore, MMP activity and the resultant destruction of the lungs in COPD very likely encourage epithelial cells towards a more tumourigenic phenotype. The physical attribute of emphysema pathology alone is likely to contribute to the permissible environment that encourages tumour growth and development. In fact, areas of visual emphysema are independently associated with lung tumour development [12].

**Fig. 2:**
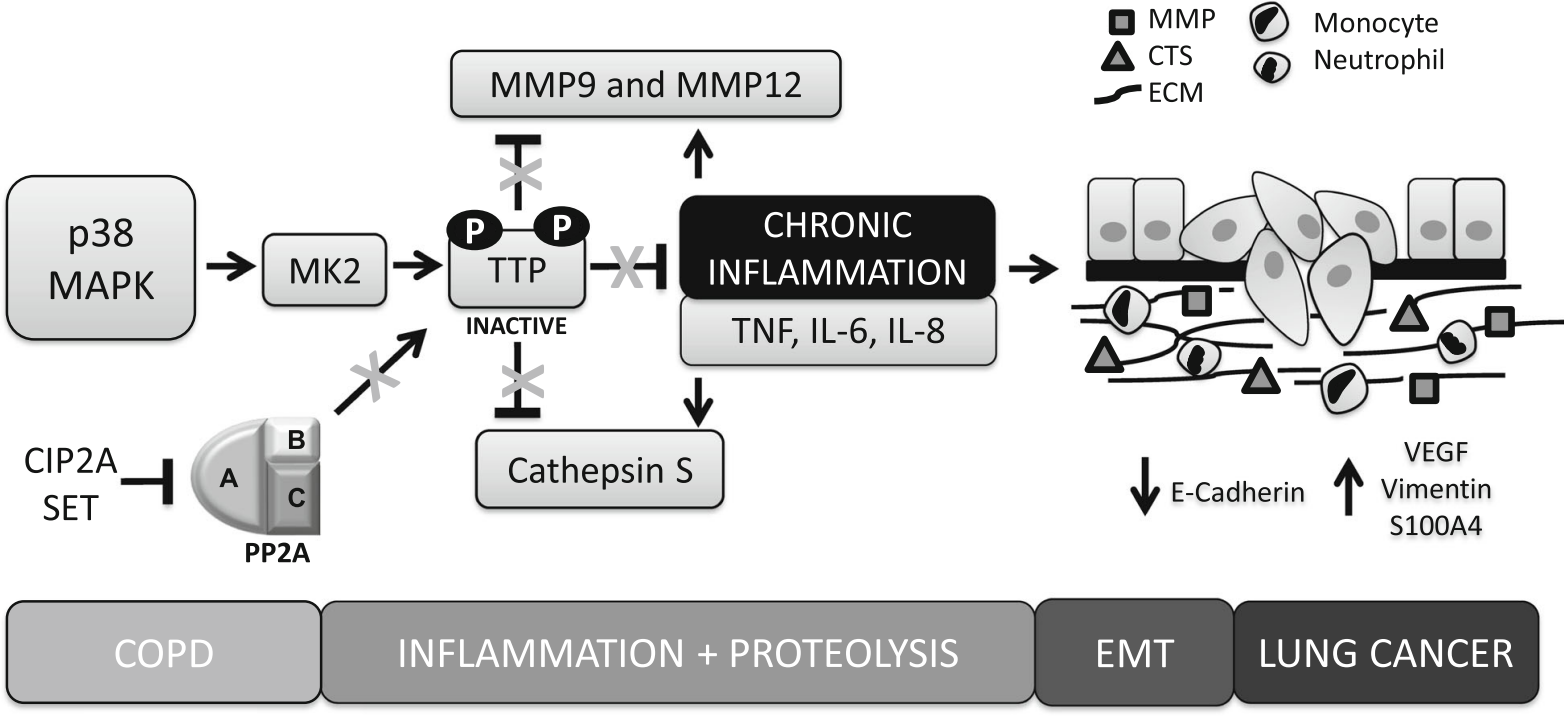
COPD creates a favourable microenvironment for lung cancer initiation and progression. During COPD the p38 MAPK pathway is activated. P38 MAPK activates MK2, subsequently mediating the phosphorylation and thereby the inactivation of TTP. Therefore, TTP is unable to degrade the mRNA of proinflammatory cytokines (TNF, IL-6, IL-8), metalloproteinases 9 and 12 (MMP9 and MMP12), and cathepsin S (CTSS). Simultaneously endogenous inhibitors CIP2A and SET are upregulated and inhibit PP2A activity, reducing the dephosphorylation and thus activation of TTP. Cytokine secretion results in the recruitment of neutrophils and monocytes. All of the above collectively create a sustained chronic inflammatory environment, which feeds further secretion of MMP9, MMP12 and CTSS to promote proteolysis. These factors degrade the ECM in the lung parenchyma leading to subsequent airspace enlargement and rbm fragmentation. The expression of epithelial marker E-cadherin is decreased, whilst the expression of mesenchymal markers vimentin and S100A4 is increased, in conjunction with increased VEGF secretion. Chronic inflammation and high proteolytic activity collectively create an ideal niche for epithelial to mesenchymal transition (EMT), promoting the oncogenic transformation of resident cells of the lung, and encouraging LC initiation and progression

Collectively, PP2A dysregulation in COPD has the potential to cause a self-sustaining cycle of inflammation and destruction that ultimately promotes LC formation (Fig. 2). Chronic inflammation activates MMPs and CTSs, leading to a cascade of proteolytic degradation, which then activates cytokine secretion to promote further inflammation, and neutrophil and monocyte infiltration [148, 149].

## Reactivating PP2A: a future pharmacotherapeutic approach in COPD and lung cancer?

New therapies must be developed to improve clinical outcomes of lung disorders, in particular, those that are refractory to current conventional methods of treatments. Research efforts aimed at restoring PP2A activity to reduce chronic inflammation and inhibit tumour growth have been of growing interest in the fields of LC and COPD. The growing awareness of the impact of PP2A endogenous inhibitors in COPD and LC suggests that targeting these inhibiting agents may have beneficial therapeutic effects through the reactivation of anti-inflammatory and tumour suppressive functions of PP2A.

### Reactivating PP2A via SET inhibition

As mentioned previously, the anti-inflammatory action of PP2A, mediated by the PP2A activator FTY720, has been demonstrated in A549 lung epithelial cells as an in vitro model of COPD [32]. Moreover, FTY720 shows anti-cancer properties in multiple cancer models, at least in part by mediating PP2A reactivation, proliferation inhibition and apoptosis induction [113, 172–174]. The action of FTY720 is considered due to an interaction with SET. FTY720 treatment has been shown to inhibit SET in models of LC by directly binding to SET, resulting in the reactivation of PP2A and its subsequent tumour suppressive functions [112]. Compounds based on the FTY720 pharmacophore are therefore of clinical interest. In support are the in vitro studies have shown the apoptosis-inducing ability of FTY720 in lung malignancies [113]. A number of studies have shown the preclinical potential of FTY720 in impairing lung tumour development and progression in mouse models [112, 113, 175, 176]. FTY720 mediated inhibition of SET inhibited primary tumour growth and invasion of A549 lung cancer cell xenografts [112, 113]. This study suggests the importance of treating early-stage emphysematous COPD patients to minimise the progression and severity of LC, although this is speculative at this stage.

FTY720 therapy did not prevent the appearance of urethane-induced lung adenoma in mice, however, it did limit early tumour development during the course of treatment [176]. This suggests that FTY720 acts in a time-dependent manner with the greatest effect of FTY720 treatment being achieved through its administration during early induction of LC [175, 176].

Although FTY720 has been studied to have beneficial effect in disease models of LC and COPD, it has further been shown that FTY720 can induce sphingosine 1-phosphate (S1P) signalling responsible for the promotion of resistance of cancer cells to therapy by counteracting the pro-apoptotic effects of ceramide [177]. Elevated levels of S1P in asthma subsequently increases pro-inflammatory cytokine expression [178]. Therefore, the need to develop PP2A activators that do not have S1P agonist properties is of high interest. Alternative activators would require targeting PP2A directly for their use in anti-cancer and COPD therapy.

A chiral analog of FTY720, AAL_(S)_, has been shown to be an effective PP2A activator, significantly suppressing pro-inflammatory cytokines in cellular models of COPD [30]. AAL_(S)_ is devoid of S1P agonism and has been utilised to promote the TTP-mediated activation of PP2A in A549 cells and inhibit pro-inflammatory cytokine production [32], AAL_(S)_ has further been shown to retain the ability to activate PP2A in models of asthma and acute myeloid leukaemia [179–183]. AAL_(S)_ was shown to reduce the severity in asthma mice models by suppressing inflammation, mucus-secreting cell numbers, type 2-associated cytokines (IL-33, IL-5 and IL-13), serum IgE and airway hyper-responsiveness [181].

### Reactivating PP2A via SMAPS

Small molecule activators of PP2A (SMAP) are molecules derived from the tricyclic neuroleptics, phenothiazine and dibenzazepine and have been identified as a direct activator of PP2A [184]. SMAPs act by binding directly to the PP2A-Aα subunit and promoting the PP2A holoenzyme assembly to reactivate PP2A and alter its interactions with endogenous PP2A inhibitors [184]. The action of the SMAP compound thereby results in the inhibition of multiple oncogenic signalling pathways via the restoration of PP2A tumour suppressive functions and subsequently induces apoptosis in LC [184]. Administration of SMAP therapy to activate PP2A in KRAS-driven lung adenocarcinoma mouse xenografts resulted in the inhibition of tumour growth, decreased phosphorylation of ERK and induction of apoptosis of cancer cells when compared to vehicle controls [184]. It is important to note that tumours with mutations in the PP2A-Aα subunit exhibited resistance to SMAP therapy, indicating the regulatory subunit function of the entire PP2A holoenzyme is essential for SMAP effectivity [184]. LC patients without mutations of the PP2A holoenzyme would be best suited to receive the therapeutic benefit of SMAP therapy.

The direct activation of PP2A using SMAP therapy may further be applicable for the treatment of COPD. The administration of SMAP therapy to cigarette smoke-induced COPD mice models decreased immune cell infiltration into airways and reduced ERK phosphorylation, CTSS expression and enzyme activity when compared to the control group mice [158]. Cigarette smoke-induced inhibition of PP2A activity within the lungs was suppressed upon administration of SMAP [158]. Cigarette smoke exposed mice appeared to have less emphysematous lung destruction when treated with SMAP therapy compared to the control group and thereby had beneficial effects of improved lung function [158].

### Reactivating PP2A via CIP2A inhibition

As alluded to earlier, EGFR activation regulates CIP2A expression in COPD and in LC [28]. EGFR-TKIs have been found to induce anti-tumour effects via the inhibition of CIP2A [104, 106, 185, 186]. Erlotinib is an FDA approved drug for the second-line treatment of NSCLC. Targeting CIP2A through the use of Erlotinib has shown efficacy in EGFR mutant NSCLC [187]. Recent findings in HBE cells from COPD patients treated with Erlotinib, resulted in decreased CIP2A expression and subsequent increased PP2A activation, suggesting it may also be applicable in the treatment of COPD [28, 94, 104–106].

### Reactivating PP2A via combination therapies

There is a strong need to explore the therapeutic potential of combination therapies in the field of PP2A activators in lung disease, particularly in COPD to halt its progression to LC. It is highly plausible that the therapeutic potential of single agents targeting PP2A activators and PP2A inhibitors (mentioned above) will be improved by combination therapy. As a notable example, combination therapy involving the administration of direct activation of PP2A by SMAPS with the addition of other therapeutic agents have shown great promise in preclinical studies of COPD and LC [82, 158, 188].

As mentioned earlier, CTSS has been identified as a contributor to cigarette smoke-induced COPD by suppressing PP2A activity [158, 189]. The utilisation of CTSS enzyme inhibitors are an emerging approach in the treatment of in vitro and in vivo models of inflammatory mediated diseases [190, 191]. The potential of combination therapy of SMAPs together with CTSS inhibitors to target the PP2A/CTSS pathway may be beneficial to COPD sufferers by increasing PP2A activity and enhancing lung function [158, 189].

Acquired resistance to EGFR targeted anti-cancer tyrosine kinase inhibitors (TKIs), is often observed in LC patients due to mutations of EGFR [192, 193]. Emerging alternative anti-cancer agents have been developed to combat TKI-resistant lung adenocarcinomas [188]. Combination treatment therapy of SMAPs and second-generation EGFR-TKI, Afatinib, resulted in degradation of CIP2A via the P13K pathway in EGFR-TKI-resistant human NSCLC cell lines [188]. Enhanced effect on cell apoptosis was observed under combination treatment of SMAPs and Afatinib suggesting efficacy of the treatment in tumour growth inhibition [188].

Although the use of alternative anti-cancer agents are of promising potential, the continued development of acquired resistance to kinase inhibitors remain a major clinical challenge in the treatment of cancer. The role of PP2A inactivation in cancers may be the potential cause of emerging kinase inhibitor resistance [82]. The inhibition of PP2A in KRAS-mutant LC cells was shown to mediate MEK inhibitor resistance, highlighting the importance of functional PP2A in LC as a key modulator in cancer treatment [82]. The combination of SMAP therapy with a MEK inhibitor could overcome therapy resistance in multiple mouse models of KRAS mutant lung cancer [82]. Anti-cancer effects of tumour regression and the suppression of p-AKT and MYC was observed in K-RAS-driven LC mouse models through the administration of combination therapy of SMAPs (DT-061) and MEK inhibitor, AZD6244 [82]. The combination of PP2A activator, SMAP, with a kinase inhibitor shows promising potential in cases of therapy resistance. Better understanding of combination therapy of anti-cancer drugs along with the reactivation of PP2A via SMAPS serves as a useful platform for drug discovery and advancement in overcoming the severity of acquired drug resistance in LC.

## Summary and future directions

Patients diagnosed with COPD are at a greater risk of developing LC than those without COPD, and are more susceptible to poor treatment outcomes [2, 4]. The overlapping prevalence of COPD and LC suggest the existence of common underlying molecular mechanisms, including genetic predispositions and dysfunctional cellular responses. There is overwhelming support suggesting COPD as an instigator for LC progression, as COPD provides a favourable micro-environment for LC development. We postulate that the inactivation of PP2A (by the varied disease-related mechanisms discussed herein) creates a permissive inflammatory environment that promotes tumour initiation, and encourages growth and metastasis of the tumour post initiation.

The similarities of PP2A inactivation in both pathologies implicate PP2A as a key pathogenic link in the progression of COPD to LC. Given the critical regulatory role that PP2A plays in normal cellular functions, it is a highly plausible that a cascade of detrimental effects will result from its dysregulation and inactivation in disease settings. Accordingly, an in depth understanding of PP2A, its dysregulation and its cellular interactions associated with both diseases, is crucial for the development of therapeutics to overcome current sub-optimal treatments. Dysfunctional PP2A has been studied in both LC and COPD, but unlike in LC, little research has focussed on the mechanisms of PP2A inactivation in COPD. Given COPD precedes LC, it is crucial to understand the molecular and cellular links at this primary pathology in order to halt the progression towards LC. Additional research in the area of COPD is essential to further implicate PP2A as a mechanistic link responsible for progressing COPD towards LC pathogenesis.

If the reactivation of PP2A can be optimised, the burden of this prevalent comorbidity may be reduced, potentially halting early-stage LC development and assisting in patient survival. Research is still needed to exploit the efficacy of PP2A reactivating agents in both COPD and LC. Studies focused on the potential of PP2A reactivating agents to reduce the risk of LC formation in COPD patients will be pivotal in improving clinical outcomes for both COPD and LC patients in the future.

## Data Availability

Not applicable.

## Abbreviations

A1AT: Alpha-1-antitrypsin
AAD: Allergic airway disease
AKT: Protein kinase B
ARE: Adenosine/uridine rich elements
BORT: Bortezomib
CIP2A: Cancerous inhibitor of PP2A
COPD: Chronic obstructive pulmonary disease
CTS: Cathepsin
ECM: Extracellular matrix
EGFR: Epidermal growth factor receptor
EMT: Epithelial to mesenchymal transition
ENSA: Endosulfine alpha
ERK: Extracellular signal-regulated kinase
FTY720: Fingolimod
GEF-H1: Guanine nucleotide exchange factor H1
HBE: Human bronchial epithelial
I2PP2A or SET: Inhibitor 2 of PP2A
IPA: Ingenuity pathway analysis
JNK: Jun N-terminal Kinase
LATS2: Large tumour suppressor kinase 2
LC: Lung cancer
MAPK: Mitogen activated protein kinase
MID1: Midline 1
MK2: MAPK kinase 2
MKK4: MAPK-kinase 4
MKP-1: Mitogen activated protein kinase-1
MMP: Matrix metalloproteinase
NSCLC: Non-small cell lung cancer
p-AKT: Phosphorylated AKT
PCDH7: Protocadherin 7
PME-1: Protein phosphatase methylesterase-1
PP2A: Protein phosphatase 2A
PTP1B: Protein tyrosine phosphatase 1B
Rbm: Reticular basement membrane
RhoB: Ras homolog gene family member B
S1P: Sphingosine 1-phosphate
SCLC: Small cell lung cancer
SMAP: Small molecule PP2A activator
TCGA: The Cancer Genome Atlas
TKI: Tyrosine kinase inhibitor
TNF: Tumour necrosis factor
TRAIL: Tumour necrosis factor-related apoptosis-inducing ligand
TTP: Tristetraprolin
